# Potential Targets for Colorectal Cancer Prevention

**DOI:** 10.3390/ijms140917279

**Published:** 2013-08-22

**Authors:** Sally Temraz, Deborah Mukherji, Ali Shamseddine

**Affiliations:** Division of Hematology**/**Oncology, American University of Beirut Medical Center (AUBMC), Beirut 1107 2020, Lebanon; E-Mails: st29@aub.edu.lb (S.T.); dm25@aub.edu.lb (D.M.)

**Keywords:** chemoprevention, colorectal cancer, NSAIDs, aspirin, natural compounds, COX-2, NF-κB, survivin, IGF-1

## Abstract

The step-wise development of colorectal neoplasia from adenoma to carcinoma suggests that specific interventions could delay or prevent the development of invasive cancer. Several key factors involved in colorectal cancer pathogenesis have already been identified including cyclooxygenase 2 (COX-2), nuclear factor kappa B (NF-κB), survivin and insulin-like growth factor-I (IGF-I). Clinical trials of COX-2 inhibitors have provided the “proof of principle” that inhibition of this enzyme can prevent the formation of colonic adenomas and potentially carcinomas, however concerns regarding the potential toxicity of these drugs have limited their use as a chemopreventative strategy. Curcumin, resveratrol and quercetin are chemopreventive agents that are able to suppress multiple signaling pathways involved in carcinogenesis and hence are attractive candidates for further research.

## 1. Introduction

Colorectal cancer (CRC) is the third most diagnosed cancer in males and the second in females [1]. Although standard clinical practice necessitates screening and surveillance in the early detection of colorectal cancers, adherence to these guidelines is still low and colorectal cancer remains a lethal disease afflicting the lives of over half a million people annually [1]. Both dietary and lifestyle factors place patients at an increased risk of developing colorectal cancer. Limiting intake of processed meat and red meat by replacing them with poultry or fish as alternative protein sources and avoiding high-temperature cooking of meats is a reasonable strategy in decreasing risk of CRC [2–4]. Concerning lifestyle factors, there is compelling evidence that restricting smoking [5] and heavy alcohol consumption [6,7] as well as preventing weight gain [8] and being physically active [9,10] can have a positive influence on CRC risk.

With the limitation of population-based prevention strategies, chemoprevention by use of natural or chemical compounds that have potential to delay, prevent or reverse the development of CRC is a viable option. Among gastrointestinal cancers, esophageal and CRC are good candidates for chemoprevention due to the long precancerous stage that provides individuals with an opportunity to interfere before adenomas develop into cancers. In CRCs, individuals that are of particular interest are those with a hereditary predisposition to colorectal neoplasia such as those with familial adenomatous polyposis (FAP) or Lynch syndrome and other individuals exhibiting the aforementioned risk factors. Evidence suggests a possible role of the following agents in the development of colon cancer: Cyclooxygenase 2 (COX-2), nuclear factor kappa B (NF-κB), survivin and insulin-like growth factor-I (IGF-I). Recently, new compounds were identified that have a potential role in inhibiting one or more of these molecular agents. In this review, we provide a general overview of these molecular agents, and discuss their mechanism of action, factors that trigger their induction and potential inhibitors of these agents.

## 2. Molecular Targets for Prevention of Colorectal Tumorigenesis

### 2.1. COX-2

Three COX enzyme isoforms have been identified until now: COX-1 enzymes are expressed in normal tissue and are involved in tissue homeostasis; COX-2 enzymes are over-expressed in cases of inflammation and colorectal neoplasms; and COX-3 enzymes are variant forms of COX-1 [11]. COX-2 has been shown to play a role in the progression of tumorigenesis. This is supported by several studies that show an elevated level of COX-2 enzymes in premalignant and malignant tissue and this increase was accompanied by a decrease in the survival of cancer patients [12]. Around 40% of colorectal adenomas and 85% of CRCs are associated with an over-expression of COX-2 rendering this enzyme an attractive therapeutic target for chemoprevention [13]. COX-1 and COX-2 catalyze the rate-limiting step in the metabolic conversion of arachidonic acid (AA) to prostaglandins (PGs). PGs are involved in a variety of pathologic processes. Specifically, prostaglandin E_2_ (PGE_2_) has been shown to mediate the pro-inflammatory and tumor-promoting effects of COX-2 in CRC. The suppressor 15-prostaglandin dehydrogenase (15-PGDH) catalyzes the degradation of PGE_2_ and is down-regulated in colorectal tumorigenesis by β-catenin [14]. The actions of PGE_2_ are mediated by signaling through four distinct G-protein-coupled receptors EP_1_, EP_2_, EP_3_, and EP_4_. In cancer tissue, EP1 and EP2 mRNA expression is increased compared to normal mucosa. EP4 mRNA expression is constantly expressed in normal mucosa and cancers while the expression of EP3 mRNA is markedly decreased in colon cancer tissues. EP3 plays an important role in suppressing cell growth while its downregulation enhances colon carcinogenesis [15]. Recently, it was found that downregulation of the EP1 receptor leads to reduced Fas ligand expression which is associated with tumor growth and decreased tumor-induced immune suppression [16]. Through the EP_2_ receptor, PGE_2_ stimulates a dual signaling cascade involving activation of phosphatidylinositol 3-kinase (PI3K) and protein kinase AKT, which in turn activate the transcriptional activity of the peroxisome-proliferator-activated receptor-δ (PPAR-δ) [17]. Also, EP_2_ up-regulates the transcriptional activity of β-catenin and the activation of the P13K/AKT pathway leads to the accumulation of unphosphorylated β-catenin in the cytoplasm [18]. β-catenin then enters the nucleus whereby it activates T cell factor 4 (TCF-4) and hypoxia inducible factor-1 (HIF-1) genes that stimulate cell survival, proliferation and angiogenesis [18]. Stimulation of EP_4_ by PGE_2_ on the other hand triggers phosphorylation of the epidermal growth factor receptor (EGFR) thereby activating the P13K/AKT pathway and the RAS-mitogen-activated protein kinase (MAPK) cascade [19]. Activation of these signaling pathways promote the expression of several genes: the angiogenic vascular endothelial growth factor receptor (VEGFR), the anti-apoptotic factor B-cell lymphoma 2 (Bcl-2) and the proliferation-promoting factor cyclin D1 [20,21]. The end result is increased proliferation, migration, invasion, angiogenesis and decreased apoptosis. These findings show that an elevated level of COX-2 promotes pathogenesis and thus inhibiting this enzyme through chemoprevention could hinder the development and progression of CRC.

Inflammatory conditions provoked by a wide range of stimuli such as pathogens, nitric oxide, cytokine, some PGs, growth factors, stress factors and oncogenes could be involved in the intracellular activation of downstream COX-2 transcriptional pathways [22]. Transcriptional regulation of COX-2 gene expression can be induced by various signaling pathways. The COX-2 promoter region consists of a number of sequences that specifically bind to several transcriptional factor complexes, such as NF-κB [23–25], CCAAT/enhancer-binding protein (C/EBP) [26,27], β-catenin/T cell factor (TCF) [28–30], cAMP response element-binding protein (CREB) [27,31], nuclear factor of activated T-cells (NFAT) [32], activator protein 1 (AP-1) [33,34], PPAR [35] and HIFα [36,37]; activation of these complexes concomitantly leads to the up-regulation of COX-2 gene expression.

Following its transcription, COX-2 mRNA is trafficked into the cytoplasm where it is tightly regulated by RNA-binding proteins that promote mRNA stability, mRNA decay or translational inhibition. These RNA-binding proteins attach themselves to the AU-rich element of COX-2 mRNA where they promote their effects. The human antigen R (HuR) has been shown to positively mediate COX-2 expression in colon cancer cell lines [38] and to promote stabilization of COX-2 mRNA by protecting the poly(A) tail from degradation. In normal tissue, HuR is expressed at low levels and is localized to the nucleus, whereas in colon adenomas, adenocarcinomas and metastases, HuR is over-expressed and localized in the cytoplasm. This over-expression of HuR was coupled with an over-expression of COX-2 [39] and has been correlated with advancing stages of malignancy and poor clinical outcome [40]. This encouraged considering HuR over-expression as a general biomarker of carcinogenesis and supports the targeting of HuR as an attractive potential strategy in future therapies. CUGBP2 (CUG triplet repeat-binding protein 2) is another RNA-binding protein that stabilizes and increases the half-life of COX-2 transcripts [41]. CUGBP2 expression has been shown to be elevated during stress particularly radiation induced stress and has high affinity to COX-2 ARE leading to stabilization of COX-2 expression [41]. In normal intestinal epithelium, the COX-2 mRNA is targeted for rapid decay whereas in tumors ARE-mediated decay is compromised. TTP (tristetraprolin) opposes the action of HuR by promoting rapid decay of ARE-containing mRNAS by associating the mRNA transcript with various decay enzymes. Expression of TTP is downregulated in colon cancer whereas its delivery to colon cancer sites resulted in the down-regulation of COX-2 expression and subsequent reduction in cell growth and proliferation [39]. Hence, the presence of TTP serves as a protective strategy to control various inflammatory mediators such as COX-2, and since HuR and TTP bind a sequence region of multiple AREs that partially overlap, the down-regulation of TTP enhances HuR and results in stabilization of the transcript [42]. TIA-1 (T-Cell intracellular antigen) is an RNA-binding protein that regulates the expression of COX-2 through translational inhibition without influencing COX-2 mRNA turnover [43]. Deficiency of TIA-1 in cancer cells is associated with elevated levels of tumor necrosis factor-α (TNF-α) and COX-2 [43,44]. Although it is a transcriptional promoting protein, CUGBP2 is an inhibitor of COX-2 mRNA translation. RBM3 (RNA-binding motif protein 3), on the other hand, promotes the translation of COX-2 and is up-regulated in CRC tumors [45]. In addition, RBM3 was shown to interact with HuR and thus enhance further stability to the transcripts [46].

### 2.2. NF-κB

Elevated COX-2 expression in malignant tissues is associated with an elevated level of NF-κB that enhances the expression of the COX-2 gene. NF-κB in the blood stream is usually bound to the inhibitory protein IκB which renders it inactive. However, during an inflammatory response, the IκB kinase phosphorylates the IκB protein thus releasing NF-κB. NF-κB activation then leads other inflammatory cytokines such as TNF-α and interleukin 1 (IL-1) to bind to their receptors and become activated [47,48]. Also activation of NF-κB leads to the subsequent activation of IL-2 which can activate Janus kinase 3 (Jak3) by autophosphorylation [49]. Jak3 in turn functions as an upstream kinase that activates a signal transducer and activator of transcription 3 (Stat3). Over-expression and aberrant expression of Jak3/Stat3 have been observed in human colon cancer *in vivo* and *in vitro* and have been shown to prevent apoptosis in the tumor, leading to poor prognosis [50,51].

### 2.3. Survivin

The ability to avoid apoptosis is one of the major oncogenic switches leading to carcinogenesis [52]. Survivin belongs to the gene family of inhibitors of apoptosis proteins (IAP). It is a bifunctional regulator of mitosis and inhibitor of programmed cell death. Survivin prevents apoptosis by inhibition of caspase 3 and caspase 7, and by regulating the G2 and M phases of the cell cycle [53]. These caspases are required for the cleavage of certain proteins involved in the disassembly of the cell during apoptosis [54,55]. Heat shock protein 90 (Hsp90) is a molecular chaperone that assists the correct folding and stabilization of various proteins in cells. Hsp90 binds and stabilizes survivin [56]. Over-expression of survivin has been associated with increased drug resistance. Survivin expression is modulated via several prominent cell signalling pathways and oncogenic signalling pathways. EGFR is known to up-regulate PI3K and extracellular signal-regulated protein kinase (ERK) signalling thus leading to increased expression of HIF1-α. HIF1-α is an important transcriptional regulator of survivin expression and the inhibition of HIF1-α by RNA interference leads to decreased expression of survivin and consequent apoptosis of the SW480 cell line [57]. In the mitochondria apoptotic pathway, P53 is a tumor suppressor gene and one of the regulators of cell cycle control and apoptosis [58]. Its expression is down regulated by survivin and Bcl-2 [59]. Bcl-2 primarily mediates its antiapoptotic function by regulating cytochrome c release from mitochondria. Cytochrome c leads to activation of caspase 9 which then triggers a cascade of caspases (caspase 3, caspase 6 and caspase 7) [60]. The transcription factor p53 is mutated in most human cancers and it targets pro-apoptotic members of the Bcl-2 family. Thus, any impairment of p53 function leads to deregulation of apoptosis signaling pathways and increases tumorigenesis.

### 2.4. IGF-I

Insulin growth factor receptor 1 (IGF-R1) plays an important role in normal cell growth and differentiation. The two ligands IGF-1 and IGF-2 are able to bind and catalyze activity of IGF-R1 and both ligands have been shown to be up-regulated in cancer. IGF-1 and IGF-2 bioavailability is modulated by a family of insulin-like growth factor binding proteins (IGBPs) however IGF-2 is also controlled by the IGF-R2 [61]. The binding of IGF-2 to the IGF-R2 results in internalization and degradation of IGF-2. Binding of IGF-1 or IGF-2 to the IGF-R1 results in autophospholylation of IGF-R1 and results in the recruitment and phosphorylation of insulin receptor substrate-1 (IRS-1), IRS-2 and src-homology/collagen (Shc), all of which are known to be involved in oncogenic processes [61]. Phosphorylation of IRS-1 and IRS-2 results in activation of PI3K that subsequently activates the AKT pathway leading to activation of Bcl-2 and inhibition of p27 and BAD [62,63]. Shc binding to IGF-R1, on the other hand, leads to activation of the RAS/MAPK pathway [64]. Thus an increase of IGF-1 bioactivity has mitogenic and antiapoptotic actions on CRC cells. Insulin resistance and hyperinsulinemia lead to increased concentration of IGFs, activation of IGF receptors, activation of PI3K and Ras-Raf pathways and result in increased cell division.

## 3. Chemical Compounds with Chemopreventive Potential

### 3.1. Non-Steroidal Anti-Inflammatory Drugs (NSAIDs)

NSAIDs inhibit COX enzymes and subsequent PGE_2_ formation and action thus resulting in anti-inflammatory and anti-tumor activities. Besides inhibition of the COX enzymes, NSAIDs have been shown to stimulate 15-PGDH expression [65] and stimulate NSAID-activated gene (NAG-1) expression [66]. NAG-1 is a member of the transforming growth factor β (TGF-β) superfamily and its expression is reduced by PGE_2_ and induced by celecoxib and sulindac. Interestingly, high expression of COX-2 in human colorectal tumor tissue was related to low expression of NAG-1, suggesting a reciprocal relationship between COX-2 and NAG-1. Moreover, NSAIDs inhibit the PPAR-δ gene which is normally regulated by Adenomatous Polyposis Coli (APC) [67] and inhibit NF-κB and Jak3/Stat3 signaling and down-regulate proinflammatory cytokines to a level that inhibits inflammation and carcinogenesis [68].

#### Clinical Effects of NSAIDs on CRC

The Adenomatous Polyp PRevention on Vioxx (APPROVe) trial is a randomized controlled trial that assessed the clinical efficacy of refecoxib (a COX-2 inhibitor) on 2587 patients with a history of colorectal adenoma. Patients were randomized to receive 25 mg of refecoxib or placebo. Results showed that refecoxib use was associated with fewer adenoma recurrence (*p* < 0.001) and decreased risk of advanced adenomas (*p* < 0.01) [69]. However, refecoxib was associated with significant upper gastrointestinal events and serious thrombotic events that resulted in its removal from the market. In the Prevention of Colorectal Sporadic Adenomatous Polyps (PreSAP), investigators assessed the effect of another COX-2 inhibitor, celecoxib. The trial randomly assigned 1561 patients with previous history of adenomas to receive 400 mg of celecoxib or placebo. Results revealed that celecoxib was successful in decreasing the rate of adenomas (33.6% *versus* 49.6%, *p* < 0.001) and the rate of advanced adenomas (5.3% *versus* 10.4%, *p* < 0.001) [70]. Risk of serious cardiovascular events was 2.5% in the celecoxib group *versus* 1.9% in the placebo group. In the Adenoma Prevention with Celecoxib (APC) trial, patients with previous adenomas were assigned to 200 mg of celecoxib twice daily, 400 mg of celecoxib twice daily or to placebo. Results showed that the risk of adenomas was significantly reduced with the use of celecoxib (43.2% in the 200 mg group, 37.5% in the 400 mg group and 60.7% in placebo group). Celecoxib use was associated with serious adverse events related to cardiac toxicity particularly in patients with previous history of cardiovascular disease [71]. Due to their cardiovascular toxicity, COX-2 inhibitors are not recommended as chemopreventive agents even though they have been proven to decrease the risk of adenomas. Only celecoxib use in FAP patients has been approved as a chemopreventive agent because the benefits accrued with celecoxib use outweigh the risks involved.

### 3.2. Aspirin

**Figure 1:**
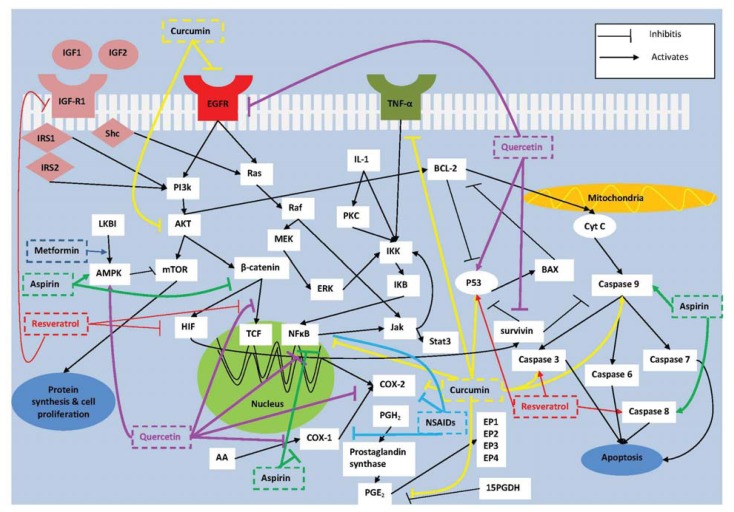
Mechanism of action of chemical compounds aspirin, metformin and NSAID (shown in green, dark blue and blue, respectively) and natural compounds curcumin, quercetin and resveratrol (shown in yellow, purple and red, respectively) in targeting pathways involved in colorectal carcinogenesis. COX-2: cyclooxygenase 2; AA: arachidonic acid; PGE_2_: prostaglandin E_2_; PGH_2_: prostaglandin H_2_; 15-PGDH: 15-prostaglandin dehydrogenase; P13K: phosphatidylinositol 3-kinase; TCF-4: T-cell factor 4; HIF-1: hypoxia inducible factor 1; MAPK: mitogen-activated protein kinase; BAX: Bcl-2 associated X protein; Bcl-2: B-cell lymphoma 2; TCF: T cell factor; Jak3: Janus kinase 3; stat3: signal transducer and activator of transcription 3; EGFR: epidermal growth factor receptor kinase; TNF-α: tissue necrosis factor α; ERK: extracellular signal-regulated protein kinase; IRS: insulin receptor substrate; Shc: Src-homology/collagen; TGF-β: transforming growth factor β; AMPK: adenosine monophosphate-activated protein kinase; mTOR: mammalian target of rapamycin; IL-1: interleukin 1; IKK: IKB kinase; IKB: inhibitor of NF-κB ; cytC: cytochrome C.

Aspirin has a short half life of 15–20 min in the human circulation and is a 50 to 100 times a potent inhibitor of COX-1 than COX-2. Aspirin targets a-nucleated platelets inducing a permanent defect in thromboxane A2 (TXA2) dependent platelet function at low doses (<75 mg/day). Thus to inhibit COX-2, high doses of aspirin are required because of the decreased sensitivity of COX-2 to aspirin [72]. Recently, aspirin has been shown to markedly inhibit thromboxane-dependent sphingosine-1-phosphate (S-1P) release from human platelets at antiplatelet doses of 100 mg/day [73]. It was shown that about 89% of human colon cancer samples stained positively for sphingosine kinase 1, the enzyme that catalyzes the formation of the biologically active lipid S-1P [74]. S-1P promotes tumor growth, neovascularization and inflammation thus giving a potential explanation to the neoplastic effects of aspirin seen at lower anti-platelet doses [75]. Aspirin additionally acetylates COX-2 switching COX-2 from synthesizing prostaglandins to antitumorigenic 15- epi-lipoxin-A4 (LXA4) or “aspirin-triggered lipoxin” (ATL) which is also seen with the use of low doses of aspirin. ATL not only acts anti-inflammatory but also inhibits proliferation of carcinoma cells [76]. To study the anti-inflammatory effect of low dose aspirin, blisters were elicited on the forearms of healthy male volunteers. Aspirin at 75 mg/day for ten days not only reduced PGE_2_ formation and white cell accumulation in inflamed tissue but also significantly increased local lipoxin production [77].Aspirin also targets COX-2 independent pathways which involve inhibition of NF-κB [78,79], and β-catenin [80] while enhancing the expression of caspases 8 and 9 [81,82] and 5′ adenosine monophosphate-activated protein kinase (AMPK) [83] (Figure 1).

#### Clinical Effects of Aspirin on CRC

The effect of aspirin on high risk populations was evaluated in 2 randomized clinical trials. In the Colorectal Adenoma/carcinoma Prevention Programme (CAPP) 1 trial, aspirin at 600 mg/day with or without resistant starch (30 g/day) was administered to 206 patients with FAP. The primary endpoint was polyp number in the rectum and sigmoid colon. Results showed that neither intervention significantly decreased the polyp number; however, there was a trend in decreased size of the largest polyp particularly with aspirin use for a period of more than 1 year (*p* = 0.02) [84]. Results from another recent double-blind, randomized clinical trial of 34 patients with FAP revealed that low dose aspirin (100 mg/day) for a period of 6–10 months resulted in a decrease in the polyp size when compared to placebo; however, the difference did not reach statistical significance. Further subgroup analysis revealed that number of subjects whose mean baseline polyp diameter was reduced to less than 2 mm was significantly higher in the aspirin group (5 subjects from aspirin group *versus* none from placebo group) [85]. In the CAPP 2 trial, aspirin at 600 mg/day and resistant starch at 30 g/day were administered to 937 patients with Lynch syndrome. In patients receiving aspirin *versus* placebo, there was no significant difference in development of advanced neoplasia between the two groups (7.4% *versus* 9.9%, *p* = 0.33) [86]. A similar result was seen in those receiving resistant starch *versus* placebo. At a median follow-up of 55.7 months, 18 patients randomly assigned to aspirin developed CRC while 30 patients assigned to resistant starch developed CRC. Aspirin at 600 mg/day for a period of 25 months is associated with a significant decrease in cancer incidence compared to placebo (*p* = 0.02) [87].

In patients with a recent history of histologically documented adenomas, the Aspirin Folate Polyp Prevention Study (AFFPS) involved 1121 randomly assigned to 325 mg aspirin/day or 81 mg aspirin/day or placebo. The incidence of one or more adenoma was 47%, 38% and 45% in the placebo, 81 mg/day group and 325 mg/day group, respectively (*p* = 0.04). After a follow up of 3 years, aspirin compared to placebo yielded a risk ratio of 0.88 for any adenoma and a risk ratio of 0.71 for advanced adenoma [88]. In the Association pour la Prévention parl’Aspirine du Cancer Colorectal (APACC), 272 patients with a history of colorectal adenomas were given 160 mg or 320 mg aspirin daily or placebo. After a follow up of 4 years, the incidence of at least one adenoma was seen in 30% in the aspirin group *versus* 41% in the placebo (*p* = 0.08). Adenomas of >5 mm diameter were seen in 10% of patients on aspirin compared to 23% of patients in the placebo group (*p* = 0.01) [89]. The Cancer and Leukemia Group B (CALGB) assessed the effect of daily aspirin 300 mg *versus* placebo in patients with previous history of CRC. After a follow up of 12.8 months, patients in the aspirin group that had at least one adenoma were 17% *versus* 27% in the placebo group (*p* = 0.004). The adjusted relative risk for adenoma recurrence in the aspirin group compared to placebo was 0.65 and the time to detect the first adenoma was significantly longer in the aspirin group (*p* = 0.022) [90]. In the United Kingdom Colorectal Adenoma Prevention (ukCAP) trial, 945 patients with a previous history of colorectal adenoma were randomized to receive 300 mg/day of aspirin or 0.5 mg of folate/day. Results revealed that 22.8% of patients receiving aspirin had recurrent adenoma compared to 28.9% in the placebo group and 9.4% in the aspirin group developed advanced adenoma compared to 15% in the placebo group. Folate, on the other hand, did not have an effect on adenoma recurrence [91]. A meta analysis of all 4 trials revealed that aspirin use at any dose was associated with a 6.7% reduction in absolute risk of adenoma and is effective in the prevention of CRC in patients with a previous history of colorectal adenomas [92].

A pooled analysis of 14,033 patient data from 5 randomized trials revealed that aspirin decreased the 20-year risk of developing colon cancer by 34% (*p* = 0.02) but not rectal cancer. Also, aspirin was capable of decreasing the risk in the proximal colon not the distal colon and doses of aspirin higher than 75 mg did not add any additional benefit [93]. Recently, an extended analysis of 51 randomized trials by the same group revealed that aspirin reduced the risk of cancer deaths by 15% and the risk of non-vascular deaths by 13% [94]. Those results were seen after 3 years of using doses of >300 mg of aspirin and after 5 years with doses <300 mg. However, it must be noted that these results are effective in tumors expressing COX-2, for tumors in the right colon and in patients taking aspirin for a duration exceeding 10 years [93,95].

### 3.3. Metformin

Obesity is a pro-inflammatory state characterized by elevated cytokines and has been shown to be involved with higher levels of COX-2 expression when compared to people with a normal body mass index [96]. Obesity is also associated with insulin resistance and hyperinsulinemia which are associated with colon cancer pathogenesis [97]. Specifically, insulin is capable of elevating levels of IGF-1 either directly or by decreasing the IGF binding proteins thus resulting in free IGF-1 levels. IGF-1 then binds to its receptor where it activates downstream signaling pathways that are involved in CRC. C-peptide is a marker of insulin secretion which has been shown to be elevated in CRC in multiple studies and has been associated with increased risk of CRC [98,99]. Metformin is an anti-diabetic drug which has been shown to decrease the risk of colon cancer by targeting one of IGF-1s downstream pathways. Its role in inhibiting tumorigenesis is related to increased expression of AMPK that subsequently inhibits the action of mammalian target of rapamycin (mTOR) thereby inhibiting tumor growth and proliferation (Figure 1) [100]. Also, metformin could downregulate the levels of circulating insulin thus decreasing its effect on elevation of IGF-1 levels.

#### Clinical Effects of Metformin on CRC

A meta-analysis of 5 observational trials that included 108,161 patients with diabetes revealed that metformin significantly reduced the risk of colorectal neoplasms (*p* < 0.001) and the risk of CRC (*p* = 0.002) [101]. Further investigation from a short clinical trial studied the effect of metformin on rectal aberrant crypt foci (ACF), an endoscopic surrogate marker of CRC. Twenty-six non-diabetic subjects with ACF were randomized to receive metformin (*n* = 12) at a dose of 250 mg/day or no treatment (*n* = 14). After 1 month, metformin significantly reduced the mean number of ACF per patient (*p* = 0.007) [102]. This trial provides preliminary evidence of the chemopreventive role of the diabetic drug metformin. Further clinical trials are ongoing to determine its effect on colorectal polyps [103].

## 4. Natural Compounds with Chemopreventive Potential

### 4.1. Curcumin

Curcumin is a phytochemical derived from the spice turmeric and has a potential in decreasing inflammation and inhibiting the growth of neoplastic cells through cell cycle arrest and promoting apoptosis by activating the mitochondria-mediated pathway. Curcumin has many potential targets (Figure 1). Both survivin and IGF-1 play important roles in inhibiting the mitochondria-mediated pathway that results in inhibition of apoptosis and prolonged survival of colon cancer cells. Curcumin counteracts this phenomenon by activating the expression of p53 and reducing TNF-α levels thus resulting in apoptotic signal activation [104]. It also decreases mitochondrial membrane potential and activates caspases 3 and 9 thus inducing apoptosis through the mitochondria pathway [105]. Curcumin decreases the translocation of β-catenin to cytoplasm and/or nucleus. Also, it induces mitotic arrest at the G2/M stages of cell cycle characterized by spindle abnormalities, defects in chromosomes and DNA damage [106]. Moreover, curcumin has a role in anti-inflammation where it targets and inhibits COX-2 gene expression, nitric oxide synthase, NF-κB and PGE_2_ [107–110]. It also inhibits colon cancer cell growth through suppressing gene expression of EGFR by reducing the activity of transcription factor Egr-1 and modulates Akt/mTOR signaling [111,112]. Thus, curcumin is an attractive chemopreventive natural agent with multiple targets and no reported toxic or adverse events.

In one pharmacodynamic and pharmacokinetic study of oral Curcuma extract, 15 patients with advanced CRC for whom no further treatment was available were given Curcuma extract at doses between 440 and 2200 mg/day. Curcuma extract containing 36–180 mg of curcumin was given for 4 months. The dose was well tolerated with no toxicity observed. Ingestion of 440 mg for 29 days was associated with a 59% decrease in lymphocytic glutathione *S*-transferase activity. This effect was not seen at higher doses. Five patients demonstrated radiologically stable disease for 2–4 months. Despite the results of this study, it is difficult to come up with a conclusion to whether curcumin at this dose is an effective chemopreventive agent in CRC due to the small number of subjects [113]. In another dose escalation study, daily curcumin was administered at doses of 0.45–3.6 g for a period of 4 months to 15 CRC patients refractory to standard chemotherapies. A daily dose of 3.6 g of curcumin was associated with a 62% and 57% decrease in inducible PGE_2_ on days 1 and 29, respectively (*p* < 0.05) [114]. In another study, curcumin capsules were given to patients with CRC at 3 doses (3600, 1800 and 450 mg) daily for 7 days. Trace levels of curcumin were found outside the circulation. Levels of M_1_G, a marker of DNA adduct formation, were significantly decreased with the curcumin dose of 3600 mg. The study concluded that curcumin at daily doses of 3.6 g is pharmacologically efficacious [115]. In a phase IIa clinical trial, curcumin at a dose of 2 g or 4 g was administered over a 30-day period to 44 eligible smokers with 8 or more ACF. Results showed that curcumin at a dose of 4 g significantly reduced the ACF number by 40% (*p* < 0.005) whereas the ACF number was not reduced by the 2 g dose [116]. Recently, curcumin was administered to 126 patients with CRC after diagnosis and before undergoing surgery. Patients either received 360 mg of curcumin 3 times daily or placebo 3 times daily. Results showed that curcumin treatment was associated with increased body weight, decreased serum TNF-α levels, enhanced p53 expression and modulated tumor cell apoptosis [104]. Other ongoing clinical trials of curcumin are listed in Table 1.

Major research with regards to curcumin has been targeted at improving its bioavailability. Poor bioavailability has been attributed to reduced absorption and rapid metabolism and elimination [117]. Low levels of serum and plasma curcumin levels have been reported. In one human clinical trial, 3.6 g of curcumin orally produced plasma curcumin levels of 11.1 nmol/L after 1 h of dosing [114]. 10 or 12 g/mL of curcumin administered orally showed curcumin levels in serum to be around 50 ng/mL; however, this resulted in minimum availability of curcumin in blood circulation to achieve its therapeutic effects [118]. In one recent clinical pilot study, curcumin C3 complex (2.35 g) was administered to 24 patients once daily for 14 days before endoscopic biopsy or colonic resection. Curcumin was detectable in the gastrointestinal tract of all patients recruited and levels of the parent compound in colonic tissue were 48.4 ± 20.9 μg/g without prior washing. Pharmacologically active levels of curcumin were recovered from the bowel mucosa even after multiple tissue washes, and there was no systemic curcumin accumulation [119]. Some of the possible ways to overcome the issue of bioavailability is through the use of piperine or through delivery of active curcumin to tumors through the use of nanoparticles, liposomes, micelles, phospholipid complexes and other formulations [120].

### 4.2. Resveratrol

Resveratrol is a polyphenol derived from grapes, berries and other plant sources. It is a natural compound that aids in suppressing the risk of obesity induced cancer. It does so through the suppression of IGF-R1 protein levels and consequently attenuates the AKT/wingless (wnt) signaling pathways (Figure 1). Another mechanism by which resveratrol results in decreased wnt signaling is through disruption of β-catenin and TCF binding and down regulating HIF-1α levels [121,122]. Resveratrol induced apoptosis of human colon cancer cells by enhancing the activation of tumor suppressor p53 that ultimately leads to activation of apoptosis [123]. It also activates caspases 3 and 8 and increases the BAX/Bcl-2 ratio. However the authors suggest that the cytotoxic effects of resveratrol are enhanced through calorie restriction [124]. The process by which resveratrol enhances the cleavage of caspases 3 and 8 is through reactive oxygen species [125]. Resveratrol has a role in suppressing inflammatory responses through decreasing nitric oxide levels and inhibiting the phosphorylation of the IKB complex thus interfering with the activation of NF-κB dependent mechanisms [126].

Preclinical trials have shown that resveratrol is efficacious in targeting pathways involved in colorectal carcinogenesis. Major clinical pilot studies thereafter have been exclusively aimed at defining the pharmacokinetics and metabolism of resveratrol since doses employed in rodent models have been much higher than doses which can be ingested with diet. In a pilot study, resveratrol at repeated daily doses of up to 5 g for a period of 29 days revealed that it is safe, only reversible diarrhea was seen in some patients at the 2.5 g and 5 g doses [127]. In a clinical trial of 20 patients with CRC, resveratrol was given at 0.5 g of 1.0 g doses orally for 8 days prior to surgery. Resveratrol was well tolerated. Resveratrol and its metabolites were detected in CRC resection tissue. The results revealed that resveratrol at doses of 0.5 g or 1.0 g reduced tumor cell proliferation by 5% (*p* = 0.005) and are enough to induce anticarcinogenic effects in colon tumors [128]. Resveratrol as a single agent at a dose of 5 g generated peak plasma concentrations of 2.4 nmol/mL [129], a level (about 10 nmol/mL) below that needed for resveratrol to elicit its chemopreventive effects in cells *in vitro* [130]. Moreover, levels of metabolic resveratrol conjugates exceeded the parent agent by about 6-fold [129]. Hence, the bioavailability of resveratrol as a single agent or as part of a dietary mixture has been the focus of many clinical studies. In a recent study evaluating colorectal tissue concentrations of resveratrol in patients with colorectal cancer prior to surgery, resveratrol concentrations of 18.6 and 674 nmol/mL were observed at 0.5 g and 1.0 g dose levels. Moreover, parent resveratrol accounted for a much larger proportion of resveratrol species in colorectal tissue than in plasma. These observations support the notion that the colorectum is a suitable target for chemoprevention by resveratrol and 0.5 g and 1.0 g of resveratrol have the capacity to elicit pharmacological effects in the gastrointestinal tract [128]. Recently, to overcome limitations of the low systemic availability of resveratrol due to the rapid and extensive metabolism, a micronized form of resveratrol (SRT501) was administered at 5.0 g dose for 14 days to patients with CRC and hepatic metastases. Mean plasma doses of resveratrol exceeded those published with equivalent doses of non-micronized resveratrol. Caspase 3 levels were increased by 39% in malignant hepatic tissue [131].

### 4.3. Quercetin

Quercetin is a flavonoid and polyphenol found in many fruits and vegetables. Quercetin has been shown to have a role in inhibiting tumorigenesis in colon cells through anti-inflammatory as well as pro-apoptotic mechanism (Figure 1). Quercetin inhibits COX-2 and COX-1 gene expression and down-regulates Bcl-2 through inhibition of NF-κB [132,133]. Quercetin induces apoptosis via up-regulation of p53 and AMPK signaling [134] and suppresses phosphorylation of EGFR thus inhibiting downstream signaling in colon carcinoma cells [135]. Quercetin decreases cell growth by disrupting the binding of β-catenin to TCF-4 and inducing G2/M cell cycle arrest through decreasing gene expression of survivin and cyclin D [136,137]. Clinical trials of quercetin effect on CRC are lacking. Evidence of efficacy of quercetin comes from preclinical studies. Thus, further research in this field is warranted and inclusion of quercetin in clinical trials is recommended as a single agent or in combination as discussed next.

Concern regarding the use of dietary sources of quercetin have become clear in recent years due to its complex intestinal absorption and poor bioavailability. Following gut biochemical modification and absorption, quercetin bioavailability *in vivo* reaches nanomolar ranges of plasma concentration (<100 nM) which is much lower than the micromolar ranges effective in *in vitro* studies [138]. Hence, to improve its bioavailability, multiple approaches have been undertaken than involve improved drug delivery systems such as inclusion complexes, liposomes, nanoparticles or micelles, which appear to provide higher solubility and bioavailability [139]. In one pharmacokinetic study in rats, quercetin was administered in a solid, lipid nanoparticle as an oral delivery carrier that resulted in a 5.7 fold increase in its bioavailability [140]. Enhanced bioavailability of quercetin in the near future is likely to bring this agent to the forefront of chemopreventive agents used in colon cancer.

## 5. Combination Therapy

Aspirin and other NSAIDs have been tested in randomized clinical trials and have been successful in reducing the risk of CRC. Unfortunately, by inhibiting COX enzymes, they tend to inhibit physiologically important PGs that could ultimately lead to potentially fatal toxicities that exclude their long-term use for cancer chemoprevention. COX-2 inhibitors and NSAIDS are associated with an increased risk for cardiovascular events, gastrointestinal ulcerations and bleeding. As for aspirin, even though it has a more favorable cardiovascular profile, hemorrhagic stroke and gastrointestinal bleeding are a concern particularly with prolonged use of the drug.

Many new natural compounds are emerging as potential inhibitors of colorectal tumorigenesis. Besides having a role in inhibiting certain effector pathways that are involved in cancer development, these compounds do not have the toxicity profile associated with the use of NSAIDS. Specifically, compounds such as curcumin, quercetin and resveratrol have the potential to target aspirin and NSAID-specific pathways that lead to downregulation of tumorigenesis. Combination therapy is a promising field in CRC chemoprevention since combining compounds targets more pathways involved in cancer and also limits the toxicity from a high dose of a single compound as in the case of aspirin and other NSAIDs. The efficacy of combination therapy has been shown in several studies. In one study combination of aspirin with metformin led to synergism between the two drugs that resulted in greater AMPK activation than each agent alone [141]. In one study, curcumin additively inhibited the growth of CRC in a rat model; the number of ACF was lower with combined therapy than with each agent alone [142]. Moreover, in another study on CRC cells, curcumin augmented celecoxib inhibition of PGE_2_ synthesis and the combination of the two synergistically down-regulated COX-2 mRNA expression [143].

Combination therapy with natural compounds has also shown promising results. In one uncontrolled trial, the combination of curcumin and quercetin was studied in 5 patients with previous colectomy. Patients were given 480 mg of curcumin and 20 mg of quercetin orally 3 times per day for a period of 3–9 months. Results showed a mean 60.4% decrease in rectal and ileal polyp number from baseline and a 50.9% decrease in polyp size. The only adverse events reported were self-limited diarrhea in one patient and mild nausea and sour taste after ingestion of the pill in another patient which subsided 3 days later [144]. These results have prompted the evaluation of curcumin at doses of 1–3 g daily for 12 months in FAP patients (Table 1). Combination of curcumin with resveratrol was shown to have better antioxidative properties than each agent alone. Also, the synergism seen with curcumin and resveratrol was more than that seen when curcumin was combined with quercetin [145]. The inhibition of tumors with a curcumin/resveratrol combination was associated with the reduction in proliferation and stimulation of apoptosis accompanied by attenuation of NF-κB activity. *In vitro* studies further demonstrated that the combination therapy resulted in greater inhibition of EGFR and IGF-R1, both of which have been implicated as possible pathways in CRC development [146]. Both curcumin and resveratrol or curcumin and quercetin have shown potential for being effective chemopreventive agents. However, no study yet has included all three combinations. The involvement of all three natural compounds might have the potential to result in chemoprevention as effectively as aspirin and other NSAIDs. As mentioned previously, aspirin is capable of inhibiting NF-κB, β-catenin, and activate AMPK and caspases 8 and 9. Using resveratrol alongside curcumin targets the same pathways as those of aspirin with the exception of activating AMPK and the subsequent inhibition of mTOR. Thus combining curcumin/resveratrol with quercetin could yield the same effect as that achieved with aspirin. Furthermore, since metformin is also an activator of AMPK, adding it to curcumin/resveratrol could also yield a combination whose effects are similar to those of aspirin. Moreover, it is expected that the combination of reservatrol/curcumin/quercetin could be more efficacioius than the use of aspirin alone since also IGF-R1R is inhibited by the 3 natural compounds combined, a target not inhibited by aspirin. However, no definite conclusion could be made until the effects of the 3 natural compounds are tested and their doses adjusted in an optimum way that could yield the best possible benefits with the least possible adverse events.

## 6. Conclusions

Chemoprevention with the use of NSAIDs and aspirin is an interesting field that has shown to be of great benefit against colorectal carcinogenesis. Yet the harms inflicted by these drugs have been shown to outweigh the benefits; specifically those patients at risk of developing CRC almost overlap with those at high risk for developing cardiovascular events. Hence, research has been directed towards adjusting the dosage of these drugs or combining them with other potential candidates in such a way that they act synergistically and at the same time decrease the toxic effects associated with higher doses of single agents. Recently, many natural compounds have emerged as potential candidates that are capable of targeting pathways involved in colorectal carcinogenesis. In this review, we have discussed the mechanism by which COX-2, NF-κB, survivin and IGF-1 influence colorectal carcinogenesis by depicting the pathways involved in each and potential inhibitors of these agents.

## 7. Future Directions

Curcumin, resveratrol and quercetin are chemopreventive agents that are able to suppress multiple signaling pathways involved in carcinogenesis and hence are attractive candidates for further research. Currently, resveratrol is undergoing extensive research to determine the dose efficacy and administration form that could enhance its bioavailability and chemopreventive properties (Table 1). Once these issues are resolved, the efficacy of resveratrol on CRC needs to be assessed in further clinical trials. Curcumin, on the other hand, is currently being studied not only in chemoprevention but also in clinical trials involving chemotherapy. The chemopreventive effect of curcumin on colorectal carcinogenesis is warranted in long trials to determine its long-term efficacy. Quercetin has been proven to be efficacious in preclinical studies and the design of specific clinical trials is warranted to depict possible applications of this substance in adjuvant CRC therapy. Moreover, the field of combination therapy looks promising and deserves further assessment in clinical trials, specifically if efficient doses and forms of all three natural compounds become known. Moreover, NSAIDs and aspirin use have been widely studied in randomized studies and their routine use is not recommended thus far. Only when benefits outweigh their toxic and possibly fatal effects, will they be recommended for routine use. Research targeting possible populations in which these circumstances apply is currently underway.

## Figures and Tables

**Table 1:** Current clinical trials of curcumin and resveratrol.

Title	Design/phase	Intervention	Primary endpoint	Identifier
Study Investigating the Ability of Plant Exosomes to Deliver Curcumin to Normal and Colon Cancer Tissue	Phase I	Arm 1: curcumin alone Arm 2: curcumin with plant exosomes Arm 3: no treatment	To estimate the effect of a fixed concentration of curcumin when delivered by plant exosomes compared to oral tablets of curcumin alone	NCT01294072
Phase II A Trial of Curcumin Among Patients With Prevalent Subclinical Neoplastic Lesions (Aberrant Crypt Foci)	Phase II	Patients receive 1 of 2 doses of oral curcumin once daily	To determine mean percentage change from baseline in prostaglandin E_2_ (PGE_2_) within ACF pre and post 30 days of curcumin administration at a specified dose	NCT00365209
Curcumin for the Chemoprevention of Colorectal Cancer	Phase II	4 g curcumin daily for 4 months or placebo	Cellular proliferation and apoptosis in the colonic mucosa and COX-2 expression and activity	NCT00118989
Use of Curcumin for Treatment of Intestinal Adenomas in Familial Adenomatous Polyposis (FAP)	Randomized double blind placebo controlled trial	Curcumin 2 pills twice per day or placebo 2 pills twice per day for 12 months	To determine the tolerability and efficacy of curcumin to regress intestinal adenomas by measuring duodenal and colorectal/ileal polyp number, and polyp size in patients with FAP	NCT00927485
Curcumin in Treating Patients With Familial Adenomatous Polyposis	Randomized double blind placebo controlled trial	Curcumin 2 pills per day or placebo 2 pills per day for 12 months	To determine the tolerability and efficacy of curcumin to regress intestinal adenomas by measuring duodenal and colorectal/ileal polyp number, and polyp size in FAP patients	NCT00641147
Curcumin Biomarkers	Phase I	4 g curcumin C3 tablets daily for 30 days	To identify genes that are modified by curcumin that could be used as biomarkers in future chemoprevention studies. The study will also evaluate tolerability and toxicity	NCT01333917
Resveratrol in Treating Patients With Colorectal Cancer That Can Be Removed By Surgery	Phase I	Patients with colorectal adenocarcinomas receive resveratrol for days 1 to 8 and on day 9 undergo colorectomy. Tumor biopsy will be retrieved	To study the side effects and best dose of resveratrol in treating patients with colorectal cancer that can be removed by surgery	NCT00433576

## References

[1] Jemal A., Bray F., Center M.M., Ferlay J., Ward E., Forman D. (2011). Global cancer statistics. CA Cancer J. Clin.

[2] Norat T., Bingham S., Ferrari P., Slimani N., Jenab M., Mazuir M., Overvad K., Olsen A., Tjønneland A., Clavel F. (2005). Meat, fish, and colorectal cancer risk: The European Prospective Investigation into cancer and nutrition. J. Natl. Cancer Inst.

[3] Robertson D.J., Sandler R.S., Haile R., Tosteson T.D., Greenberg E.R., Grau M., Baron J.A. (2005). Fat, fiber, meat and the risk of colorectal adenomas. Am. J. Gastroenterol.

[4] Wu K., Giovannucci E., Byrne C., Platz E.A., Fuchs C., Willett W.C., Sinha R. (2006). Meat mutagens and risk of distal colon adenoma in a cohort of U.S. men. Cancer Epidemiol. Biomarkers Prev.

[5] Liang P.S., Chen T.Y., Giovannucci E. (2009). Cigarette smoking and colorectal cancer incidence and mortality: Systematic review and meta-analysis. Int. J. Cancer.

[6] Cho E., Smith-Warner S.A., Ritz J., van den Brandt P.A., Colditz G.A., Folsom A.R., Freudenheim J.L., Giovannucci E., Goldbohm R.A., Graham S. (2004). Alcohol intake and colorectal cancer: A pooled analysis of 8 cohort studies. Ann. Intern. Med.

[7] Ferrari P., Jenab M., Norat T., Moskal A., Slimani N., Olsen A., Tjønneland A., Overvad K., Jensen M.K., Boutron-Ruault M.C. (2007). Lifetime and baseline alcohol intake and risk of colon and rectal cancers in the European prospective investigation into cancer and nutrition (EPIC). Int. J. Cancer.

[8] Ning Y., Wang L., Giovannucci E.L. (2010). A quantitative analysis of body mass index and colorectal cancer: Findings from 56 observational studies. Obes. Rev.

[9] Colbert L.H., Hartman T.J., Malila N., Limburg P.J., Pietinen P., Virtamo J., Taylor P.R., Albanes D. (2001). Physical activity in relation to cancer of the colon and rectum in a cohort of male smokers. Cancer Epidemiol. Biomarkers Prev.

[10] Colditz G.A., Cannuscio C.C., Frazier A.L. (1997). Physical activity and reduced risk of colon cancer: Implications for prevention. Cancer Causes Control.

[11] Hahn E., Kraus S., Arber N. (2010). Role of cyclooxygenase-2 in pathogenesis and prevention of colorectal cancer. Dig. Dis.

[12] Wang D., Dubois R.N. (2010). The role of COX-2 in intestinal inflammation and colorectal cancer. Oncogene.

[13] Eberhart C.E., Coffey R.J., Radhika A., Giardiello F.M., Ferrenbach S., DuBois R.N. (1994). Up-regulation of cyclooxygenase 2 gene expression in human colorectal adenomas and adenocarcinomas. Gastroenterology.

[14] Smartt H.J., Greenhough A., Ordóñez-Morán P., Talero E., Cherry C.A., Wallam C.A., Parry L., Al Kharusi M., Roberts H.R., Mariadason J.M. (2012). β-catenin represses expression of the tumour suppressor 15-prostaglandin dehydrogenase in the normal intestinal epithelium and colorectal tumour cells. Gut.

[15] Shoji Y., Takahashi M., Kitamura T., Watanabe K., Kawamori T., Maruyama T., Sugimoto Y., Negishi M., Narumiya S., Sugimura T. (2004). Downregulation of prostaglandin E receptor subtype EP3 during colon cancer development. Gut.

[16] O’Callaghan G., Ryan A., Neary P., O’Mahony C., Shanahan F., Houston A. (2013). Targeting the EP1 receptor reduces Fas ligand expression and increases the antitumor immune response in an *in vivo* model of colon cancer. Int. J. Cancer.

[17] Wang D., Wang H., Shi Q., Katkuri S., Walhi W., Desvergne B., Das S.K., Dey S.K., DuBois R.N. (2004). Prostaglandin E(2) promotes colorectal adenoma growth via transactivation of the nuclear peroxisome proliferator-activated receptor delta. Cancer Cell.

[18] Buchanan F.G., DuBois R.N. (2006). Connecting COX-2 and Wnt in cancer. Cancer Cell.

[19] Pai R., Soreghan B., Szabo I.L., Pavelka M., Baatar D., Tarnawski A.S. (2002). Prostaglandin E2 transactivates EGF receptor: A novel mechanism for promoting colon cancer growth and gastrointestinal hypertrophy. Nat. Med.

[20] Fukuda R., Kelly B., Semenza G.L. (2003). Vascular endothelial growth factor gene expression in colon cancer cells exposed to prostaglandin E2 is mediated by hypoxia-inducible factor 1. Cancer Res.

[21] Sheng H., Shao J., Morrow J.D., Beauchamp R.D., DuBois R.N. (1998). Modulation of apoptosis and Bcl-2 expression by prostaglandin E2 in human colon cancer cells. Cancer Res.

[22] Tanabe T., Tohnai N. (2002). Cyclooxygenase isozymes and their gene structures and expression. Prostaglandins Other Lipid Mediat..

[23] Kojima M., Morisaki T., Izuhara K., Uchiyama A., Matsunari Y., Katano M., Tanaka M. (2000). Lipopolysaccharide increases cyclo-oxygenase-2 expression in a colon carcinoma cell line through nuclear factor-kappa B activation. Oncogene.

[24] Schmedtje J.F., Ji Y.S., Liu W.L., DuBois R.N., Runge M.S. (1997). Hypoxia induces cyclooxygenase-2 via the NF-kappaB p65 transcription factor in human vascular endothelial cells. J. Biol. Chem.

[25] Singer I.I., Kawka D.W., Schloemann S., Tessner T., Riehl T., Stenson W.F. (1998). Cyclooxygenase 2 is induced in colonic epithelial cells in inflammatory bowel disease. Gastroenterology.

[26] Kim Y., Fischer S.M. (1998). Transcriptional regulation of cyclooxygenase-2 in mouse skin carcinoma cells. Regulatory role of CCAAT/enhancer-binding proteins in the differential expression of cyclooxygenase-2 in normal and neoplastic tissues. J. Biol. Chem.

[27] Shao J., Sheng H., Inoue H., Morrow J.D., DuBois R.N. (2000). Regulation of constitutive cyclooxygenase-2 expression in colon carcinoma cells. J. Biol. Chem.

[28] Araki Y., Okamura S., Hussain S.P., Nagashima M., He P., Shiseki M., Miura K., Harris C.C. (2003). Regulation of cyclooxygenase-2 expression by the Wnt and ras pathways. Cancer Res.

[29] Howe L.R., Subbaramaiah K., Chung W.J., Dannenberg A.J., Brown A.M. (1999). Transcriptional activation of cyclooxygenase-2 in Wnt-1-transformed mouse mammary epithelial cells. Cancer Res.

[30] Mei J.M., Hord N.G., Winterstein D.F., Donald S.P., Phang J.M. (1999). Differential expression of prostaglandin endoperoxide H synthase-2 and formation of activated beta-catenin-LEF-1 transcription complex in mouse colonic epithelial cells contrasting in Apc. Carcinogenesis.

[31] Subbaramaiah K., Cole P.A., Dannenberg A.J. (2002). Retinoids and carnosol suppress cyclooxygenase-2 transcription by CREB-binding protein/p300-dependent and -independent mechanisms. Cancer Res.

[32] Hernández G.L., Volpert O.V., Iñiguez M.A., Lorenzo E., Martínez-Martínez S., Grau R., Fresno M., Redondo J.M. (2001). Selective inhibition of vascular endothelial growth factor-mediated angiogenesis by cyclosporin A: Roles of the nuclear factor of activated T cells and cyclooxygenase 2. J. Exp. Med.

[33] Miller C., Zhang M., He Y., Zhao J., Pelletier J.P., Martel-Pelletier J., Di Battista J.A. (1998). Transcriptional induction of cyclooxygenase-2 gene by okadaic acid inhibition of phosphatase activity in human chondrocytes: Co-stimulation of AP-1 and CRE nuclear binding proteins. J. Cell. Biochem.

[34] Subbaramaiah K., Norton L., Gerald W., Dannenberg A.J. (2002). Cyclooxygenase-2 is overexpressed in HER-2/neu-positive breast cancer: Evidence for involvement of AP-1 and PEA3. J. Biol. Chem.

[35] Meade E.A., McIntyre T.M., Zimmerman G.A., Prescott S.M. (1999). Peroxisome proliferators enhance cyclooxygenase-2 expression in epithelial cells. J. Biol. Chem.

[36] Bazan N.G., Lukiw W.J. (2002). Cyclooxygenase-2 and presenilin-1 gene expression induced by interleukin-1beta and amyloid beta 42 peptide is potentiated by hypoxia in primary human neural cells. J. Biol. Chem.

[37] Kaidi A., Qualtrough D., Williams A.C., Paraskeva C. (2006). Direct transcriptional up-regulation of cyclooxygenase-2 by hypoxia-inducible factor (HIF)-1 promotes colorectal tumor cell survival and enhances HIF-1 transcriptional activity during hypoxia. Cancer Res.

[38] Dixon D.A., Tolley N.D., King P.H., Nabors L.B., McIntyre T.M., Zimmerman G.A., Prescott S.M. (2001). Altered expression of the mRNA stability factor HuR promotes cyclooxygenase-2 expression in colon cancer cells. J. Clin. Invest.

[39] Young L.E., Sanduja S., Bemis-Standoli K., Pena E.A., Price R.L., Dixon D.A. (2009). The mRNA binding proteins HuR and tristetraprolin regulate cyclooxygenase 2 expression during colon carcinogenesis. Gastroenterology.

[40] Dixon D.A. (2003). Regulation of COX-2 expression in human cancers. Prog. Exp. Tumor Res.

[41] Mukhopadhyay D., Houchen C.W., Kennedy S., Dieckgraefe B.K., Anant S. (2003). Coupled mRNA stabilization and translational silencing of cyclooxygenase-2 by a novel RNA binding protein, CUGBP2. Mol. Cell.

[42] Anant S., Houchen C.W. (2009). HuR and TTP: Two RNA binding proteins that deliver message from the 3′ end. Gastroenterology.

[43] Dixon D.A., Balch G.C., Kedersha N., Anderson P., Zimmerman G.A., Beauchamp R.D., Prescott S.M. (2003). Regulation of cyclooxygenase-2 expression by the translational silencer TIA-1. J. Exp. Med.

[44] Piecyk M., Wax S., Beck A.R., Kedersha N., Gupta M., Maritim B., Chen S., Gueydan C., Kruys V., Streuli M. (2000). TIA-1 is a translational silencer that selectively regulates the expression of TNF-alpha. EMBO J.

[45] Sureban S.M., Ramalingam S., Natarajan G., May R., Subramaniam D., Bishnupuri K.S., Morrison A.R., Dieckgraefe B.K., Brackett D.J., Postier R.G. (2008). Translation regulatory factor RBM3 is a proto-oncogene that prevents mitotic catastrophe. Oncogene.

[46] Anant S., Houchen C.W., Pawar V., Ramalingam S. (2010). Role of RNA-binding proteins in colorectal carcinogenesis. Curr. Colorectal Cancer Rep.

[47] Poligone B., Baldwin A.S. (2001). Positive and negative regulation of NF-kappaB by COX-2: Roles of different prostaglandins. J. Biol. Chem.

[48] Yasui H., Adachi M., Imai K. (2003). Combination of tumor necrosis factor-alpha with sulindac augments its apoptotic potential and suppresses tumor growth of human carcinoma cells in nude mice. Cancer.

[49] Cornejo M.G., Boggon T.J., Mercher T. (2009). JAK3: A two-faced player in hematological disorders. Int. J. Biochem. Cell Biol.

[50] Lin Q., Lai R., Chirieac L.R., Li C., Thomazy V.A., Grammatikakis I., Rassidakis G.Z., Zhang W., Fujio Y., Kunisada K. (2005). Constitutive activation of JAK3/STAT3 in colon carcinoma tumors and cell lines: Inhibition of JAK3/STAT3 signaling induces apoptosis and cell cycle arrest of colon carcinoma cells. Am. J. Pathol.

[51] Tsareva S.A., Moriggl R., Corvinus F.M., Wiederanders B., Schütz A., Kovacic B., Friedrich K. (2007). Signal transducer and activator of transcription 3 activation promotes invasive growth of colon carcinomas through matrix metalloproteinase induction. Neoplasia.

[52] Qiao L., Wong B.C. (2009). Targeting apoptosis as an approach for gastrointestinal cancer therapy. Drug Resist. Updat.

[53] Mita A.C., Mita M.M., Nawrocki S.T., Giles F.J. (2008). Survivin: Key regulator of mitosis and apoptosis and novel target for cancer therapeutics. Clin. Cancer Res.

[54] Schimmer A.D. (2004). Inhibitor of apoptosis proteins: Translating basic knowledge into clinical practice. Cancer Res.

[55] Yang Y.L., Li X.M. (2000). The IAP family: Endogenous caspase inhibitors with multiple biological activities. Cell Res.

[56] Cheung C.H., Chen H.H., Cheng L.T., Lyu K.W., Kanwar J.R., Chang J.Y. (2010). Targeting Hsp90 with small molecule inhibitors induces the over-expression of the anti-apoptotic molecule, survivin, in human A549, HONE-1 and HT-29 cancer cells. Mol. Cancer.

[57] Wu X.Y., Fu Z.X., Wang X.H. (2010). Effect of hypoxia-inducible factor 1-α on Survivin in colorectal cancer. Mol. Med. Rep.

[58] Waldman T., Kinzler K.W., Vogelstein B. (1995). p21 is necessary for the p53-mediated G1 arrest in human cancer cells. Cancer Res.

[59] Huerta S., Goulet E.J., Livingston E.H. (2006). Colon cancer and apoptosis. Am. J. Surg.

[60] Acehan D., Jiang X., Morgan D.G., Heuser J.E., Wang X., Akey C.W. (2002). Three-dimensional structure of the apoptosome: Implications for assembly, procaspase-9 binding, and activation. Mol. Cell.

[61] Zha J., Lackner M.R. (2010). Targeting the insulin-like growth factor receptor-1R pathway for cancer therapy. Clin. Cancer Res.

[62] Guertin D.A., Sabatini D.M. (2005). An expanding role for mTOR in cancer. Trends Mol. Med.

[63] Datta S.R., Brunet A., Greenberg M.E. (1999). Cellular survival: A play in three Akts. Genes Dev.

[64] Pollak M. (2008). Insulin and insulin-like growth factor signalling in neoplasia. Nat. Rev. Cancer.

[65] Tai H.H., Chi X., Tong M. (2011). Regulation of 15-hydroxyprostaglandin dehydrogenase (15-PGDH) by non-steroidal anti-inflammatory drugs (NSAIDs). Prostaglandins Other Lipid Mediat.

[66] Iguchi G., Chrysovergis K., Lee S.H., Baek S.J., Langenbach R., Eling T.E. (2009). A reciprocal relationship exists between non-steroidal anti-inflammatory drug-activated gene-1 (NAG-1) and cyclooxygenase-2. Cancer Lett.

[67] He T.C., Chan T.A., Vogelstein B., Kinzler K.W. (1999). PPARdelta is an APC-regulated target of nonsteroidal anti-inflammatory drugs. Cell.

[68] Vaish V., Sanyal S.N. (2011). Chemopreventive effects of NSAIDs on cytokines and transcription factors during the early stages of colorectal cancer. Pharmacol. Rep.

[69] Baron J.A., Sandler R.S., Bresalier R.S., Quan H., Riddell R., Lanas A., Bolognese J.A., Oxenius B., Horgan K., Loftus S. (2006). A randomized trial of rofecoxib for the chemoprevention of colorectal adenomas. Gastroenterology.

[70] Arber N., Eagle C.J., Spicak J., Rácz I., Dite P., Hajer J., Zavoral M., Lechuga M.J., Gerletti P., Tang J. (2006). Celecoxib for the prevention of colorectal adenomatous polyps. N. Engl. J. Med.

[71] Bertagnolli M.M., Eagle C.J., Zauber A.G., Redston M., Solomon S.D., Kim K., Tang J., Rosenstein R.B., Wittes J., Corle D. (2006). Celecoxib for the prevention of sporadic colorectal adenomas. N. Engl. J. Med.

[72] Patrono C., García Rodríguez L.A., Landolfi R., Baigent C. (2005). Low-dose aspirin for the prevention of atherothrombosis. N. Engl. J. Med.

[73] Ulrych T., Böhm A., Polzin A., Daum G., Nüsing R.M., Geisslinger G., Hohlfeld T., Schrör K., Rauch B.H. (2011). Release of sphingosine-1-phosphate from human platelets is dependent on thromboxane formation. J. Thromb. Haemost.

[74] Kawamori T., Kaneshiro T., Okumura M., Maalouf S., Uflacker A., Bielawski J., Hannun Y.A., Obeid L.M. (2009). Role for sphingosine kinase 1 in colon carcinogenesis. FASEB J.

[75] Pyne N.J., Pyne S. (2010). Sphingosine 1-phosphate and cancer. Nat. Rev. Cancer.

[76] Clària J., Lee M.H., Serhan C.N. (1996). Aspirin-triggered lipoxins (15-epi-LX) are generated by the human lung adenocarcinoma cell line (A549)-neutrophil interactions and are potent inhibitors of cell proliferation. Mol. Med.

[77] Morris T., Stables M., Hobbs A., de Souza P., Colville-Nash P., Warner T., Newson J., Bellingan G., Gilroy D.W. (2009). Effects of low-dose aspirin on acute inflammatory responses in humans. J. Immunol.

[78] Kopp E., Ghosh S. (1994). Inhibition of NF-kappa B by sodium salicylate and aspirin. Science.

[79] Yin M.J., Yamamoto Y., Gaynor R.B. (1998). The anti-inflammatory agents aspirin and salicylate inhibit the activity of I(kappa)B kinase-beta. Nature.

[80] Bos C.L., Kodach L.L., van den Brink G.R., Diks S.H., van Santen M.M., Richel D.J., Peppelenbosch M.P., Hardwick J.C. (2006). Effect of aspirin on the Wnt/beta-catenin pathway is mediated via protein phosphatase 2A. Oncogene.

[81] Gu Q., Wang J.D., Xia H.H., Lin M.C., He H., Zou B., Tu S.P., Yang Y., Liu X.G., Lam S.K. (2005). Activation of the caspase-8/Bid and Bax pathways in aspirin-induced apoptosis in gastric cancer. Carcinogenesis.

[82] Zimmermann K.C., Waterhouse N.J., Goldstein J.C., Schuler M., Green D.R. (2000). Aspirin induces apoptosis through release of cytochrome c from mitochondria. Neoplasia.

[83] Hawley S.A., Fullerton M.D., Ross F.A., Schertzer J.D., Chevtzoff C., Walker K.J., Peggie M.W., Zibrova D., Green K.A., Mustard K.J. (2012). The ancient drug salicylate directly activates AMP-activated protein kinase. Science.

[84] Burn J., Bishop D.T., Chapman P.D., Elliott F., Bertario L., Dunlop M.G., Eccles D., Ellis A., Evans D.G., Fodde R. (2011). A randomized placebo-controlled prevention trial of aspirin and/or resistant starch in young people with familial adenomatous polyposis. Cancer Prev. Res. (Phila. ).

[85] Ishikawa H., Keiji W., Sadao S., Michihiro M., Keiji H., Tomiyo N., Ikuko T., Atsuko K., Nobuhisa G., Takashi A. (2013). Preventive effects of low-dose aspirin on colorectal adenoma growth in patients with familial adenomatous polyposis: Double-blind, randomized clinical trial. Cancer Med.

[86] Burn J., Bishop D.T., Mecklin J.P., Macrae F., Möslein G., Olschwang S., Bisgaard M.L., Ramesar R., Eccles D., Maher E.R. (2008). Effect of aspirin or resistant starch on colorectal neoplasia in the Lynch syndrome. N. Engl. J. Med.

[87] Burn J., Gerdes A.M., Macrae F., Mecklin J.P., Moeslein G., Olschwang S., Eccles D., Evans D.G., Maher E.R., Bertario L. (2011). Long-term effect of aspirin on cancer risk in carriers of hereditary colorectal cancer: An analysis from the CAPP2 randomised controlled trial. Lancet.

[88] Baron J.A., Cole B.F., Sandler R.S., Haile R.W., Ahnen D., Bresalier R., McKeown-Eyssen G., Summers R.W., Rothstein R., Burke C.A. (2003). A randomized trial of aspirin to prevent colorectal adenomas. N. Engl. J. Med.

[89] Benamouzig R., Deyra J., Martin A., Girard B., Jullian E., Piednoir B., Couturier D., Coste T., Little J., Chaussade S. (2003). Daily soluble aspirin and prevention of colorectal adenoma recurrence: One-year results of the APACC trial. Gastroenterology.

[90] Sandler R.S., Halabi S., Baron J.A., Budinger S., Paskett E., Keresztes R., Petrelli N., Pipas J.M., Karp D.D., Loprinzi C.L. (2003). A randomized trial of aspirin to prevent colorectal adenomas in patients with previous colorectal cancer. N. Engl. J. Med.

[91] Logan R.F., Grainge M.J., Shepherd V.C., Armitage N.C., Muir K.R., ukCAP Trial Group (2008). Aspirin and folic acid for the prevention of recurrent colorectal adenomas. Gastroenterology.

[92] Cole B.F., Logan R.F., Halabi S., Benamouzig R., Sandler R.S., Grainge M.J., Chaussade S., Baron J.A. (2009). Aspirin for the chemoprevention of colorectal adenomas: Meta-analysis of the randomized trials. J. Natl. Cancer Inst.

[93] Rothwell P.M., Wilson M., Elwin C.E., Norrving B., Algra A., Warlow C.P., Meade T.W. (2010). Long-term effect of aspirin on colorectal cancer incidence and mortality: 20-year follow-up of five randomised trials. Lancet.

[94] Rothwell P.M., Price J.F., Fowkes F.G., Zanchetti A., Roncaglioni M.C., Tognoni G., Lee R., Belch J.F., Wilson M., Mehta Z. (2012). Short-term effects of daily aspirin on cancer incidence, mortality, and non-vascular death: Analysis of the time course of risks and benefits in 51 randomised controlled trials. Lancet.

[95] Chan A.T., Arber N., Burn J., Chia W.K., Elwood P., Hull M.A., Logan R.F., Rothwell P.M., Schrör K., Baron J.A. (2012). Aspirin in the chemoprevention of colorectal neoplasia: An overview. Cancer Prev. Res.

[96] Delage B., Rullier A., Capdepont M., Rullier E., Cassand P. (2007). The effect of body weight on altered expression of nuclear receptors and cyclooxygenase-2 in human colorectal cancers. Nutr. J.

[97] Giovannucci E. (1995). Insulin and colon cancer. Cancer Causes Control.

[98] Ma J., Giovannucci E., Pollak M., Leavitt A., Tao Y., Gaziano J.M., Stampfer M.J. (2004). A prospective study of plasma C-peptide and colorectal cancer risk in men. J. Natl. Cancer Inst.

[99] Jenab M., Riboli E., Cleveland R.J., Norat T., Rinaldi S., Nieters A., Biessy C., Tjønneland A., Olsen A., Overvad K. (2007). Serum C-peptide, IGFBP-1 and IGFBP-2 and risk of colon and rectal cancers in the European Prospective Investigation into Cancer and Nutrition. Int. J. Cancer.

[100] Pawałowska M., Markowska A. (2012). The influence of metformin in the etiology of selected cancers. Contemp. Oncol.

[101] Zhang Z.J., Zheng Z.J., Kan H., Song Y., Cui W., Zhao G., Kip K.E. (2011). Reduced risk of colorectal cancer with metformin therapy in patients with type 2 diabetes: A meta-analysis. Diabetes Care.

[102] Hosono K., Endo H., Takahashi H., Sugiyama M., Sakai E., Uchiyama T., Suzuki K., Iida H., Sakamoto Y., Yoneda K. (2010). Metformin suppresses colorectal aberrant crypt foci in a short-term clinical trial. Cancer Prev. Res. (Phila. ).

[103] Higurashi T., Takahashi H., Endo H., Hosono K., Yamada E., Ohkubo H., Sakai E., Uchiyama T., Hata Y., Fujisawa N. (2012). Metformin efficacy and safety for colorectal polyps: A double-blind randomized controlled trial. BMC Cancer.

[104] He Z.Y., Shi C.B., Wen H., Li F.L., Wang B.L., Wang J. (2011). Upregulation of p53 expression in patients with colorectal cancer by administration of curcumin. Cancer Invest.

[105] Guo L.D., Chen X.J., Hu Y.H., Yu Z.J., Wang D., Liu J.Z. (2013). Curcumin inhibits proliferation and induces apoptosis of human colorectal cancer cells by activating the mitochondria apoptotic pathway. Phytother. Res.

[106] Blakemore L.M., Boes C., Cordell R., Manson M.M. (2013). Curcumin-induced mitotic arrest is characterized by spindle abnormalities, defects in chromosomal congression and DNA damage. Carcinogenesis.

[107] Sandur S.K., Deorukhkar A., Pandey M.K., Pabón A.M., Shentu S., Guha S., Aggarwal B.B., Krishnan S. (2009). Curcumin modulates the radiosensitivity of colorectal cancer cells by suppressing constitutive and inducible NF-kappaB activity. Int. J. Radiat. Oncol. Biol. Phys.

[108] Villegas I., Sánchez-Fidalgo S., de la Lastra C.A. (2011). Chemopreventive effect of dietary curcumin on inflammation-induced colorectal carcinogenesis in mice. Mol. Nutr. Food Res.

[109] Su C.C., Chen G.W., Lin J.G., Wu L.T., Chung J.G. (2006). Curcumin inhibits cell migration of human colon cancer colo 205 cells through the inhibition of nuclear factor kappa B/p65 and down-regulates cyclooxygenase-2 and matrix metalloproteinase-2 expressions. Anticancer Res.

[110] Lev-Ari S., Maimon Y., Strier L., Kazanov D., Arber N. (2006). Down-regulation of prostaglandin E2 by curcumin is correlated with inhibition of cell growth and induction of apoptosis in human colon carcinoma cell lines. J. Soc. Integr. Oncol.

[111] Chen A., Xu J., Johnson A.C. (2006). Curcumin inhibits human colon cancer cell growth by suppressing gene expression of epidermal growth factor receptor through reducing the activity of the transcription factor Egr-1. Oncogene.

[112] Johnson S.M., Gulhati P., Arrieta I., Wang X., Uchida T., Gao T., Evers B.M. (2009). Curcumin inhibits proliferation of colorectal carcinoma by modulating Akt/mTOR signaling. Anticancer Res.

[113] Sharma R.A., McLelland H.R., Hill K.A., Ireson C.R., Euden S.A., Manson M.M., Pirmohamed M., Marnett L.J., Gescher A.J., Steward W.P. (2001). Pharmacodynamic and pharmacokinetic study of oral Curcuma extract in patients with colorectal cancer. Clin. Cancer Res.

[114] Sharma R.A., Euden S.A., Platton S.L., Cooke D.N., Shafayat A., Hewitt H.R., Marczylo T.H., Morgan B., Hemingway D., Plummer S.M. (2004). Phase I clinical trial of oral curcumin: Biomarkers of systemic activity and compliance. Clin. Cancer Res.

[115] Garcea G., Berry D.P., Jones D.J., Singh R., Dennison A.R., Farmer P.B., Sharma R.A., Steward W.P., Gescher A.J. (2005). Consumption of the putative chemopreventive agent curcumin by cancer patients: Assessment of curcumin levels in the colorectum and their pharmacodynamic consequences. Cancer Epidemiol. Biomarkers Prev.

[116] Carroll R.E., Benya R.V., Turgeon D.K., Vareed S., Neuman M., Rodriguez L., Kakarala M., Carpenter P.M., McLaren C., Meyskens F.L. (2011). Phase IIa clinical trial of curcumin for the prevention of colorectal neoplasia. Cancer Prev. Res.

[117] Anand P., Kunnumakkara A.B., Newman R.A., Aggarwal B.B. (2007). Bioavailability of curcumin: Problems and promises. Mol. Pharm.

[118] Lao C.D., Ruffin M.T., Normolle D., Heath D.D., Murray S.I., Bailey J.M., Boggs M.E., Crowell J., Rock C.L., Brenner D.E. (2006). Dose escalation of a curcuminoid formulation. BMC Complement. Altern. Med.

[119] Irving G.R., Howells L.M., Sale S., Kralj-Hans I., Atkin W.S., Clark S.K., Britton R.G., Jones D.J., Scott E.N., Berry D.P. (2013). Prolonged biologically active colonic tissue levels of curcumin achieved after oral administration—A clinical pilot study including assessment of patient acceptability. Cancer Prev. Res.

[120] Yallapu M.M., Jaggi M., Chauhan S.C. (2012). Curcumin nanoformulations: A future nanomedicine for cancer. Drug Discov. Today.

[121] Chen H.J., Hsu L.S., Shia Y.T., Lin M.W., Lin C.M. (2012). The β-catenin/TCF complex as a novel target of resveratrol in the Wnt/β-catenin signaling pathway. Biochem. Pharmacol.

[122] Wu H., Liang X., Fang Y., Qin X., Zhang Y., Liu J. (2008). Resveratrol inhibits hypoxia-induced metastasis potential enhancement by restricting hypoxia-induced factor-1 alpha expression in colon carcinoma cells. Biomed. Pharmacother.

[123] Vanamala J., Reddivari L., Radhakrishnan S., Tarver C. (2010). Resveratrol suppresses IGF-1 induced human colon cancer cell proliferation and elevates apoptosis via suppression of IGF-1R/Wnt and activation of p53 signaling pathways. BMC Cancer.

[124] Fouad M., Agha A., Merzabani M.A., Shouman S. (2013). Resveratrol inhibits proliferation, angiogenesis and induces apoptosis in colon cancer cells: Calorie restriction is the force to the cytotoxicity. Hum. Exp. Toxicol..

[125] Miki H., Uehara N., Kimura A., Sasaki T., Yuri T., Yoshizawa K., Tsubura A. (2012). Resveratrol induces apoptosis via ROS-triggered autophagy in human colon cancer cells. Int. J. Oncol.

[126] Panaro M.A., Carofiglio V., Acquafredda A., Cavallo P., Cianciulli A. (2012). Anti-inflammatory effects of resveratrol occur via inhibition of lipopolysaccharide-induced NF-κB activation in Caco-2 and SW480 human colon cancer cells. Br. J. Nutr.

[127] Schneider Y., Duranton B., Gossé F., Schleiffer R., Seiler N., Raul F. (2001). Resveratrol inhibits intestinal tumorigenesis and modulates host-defense-related gene expression in an animal model of human familial adenomatous polyposis. Nutr. Cancer.

[128] Patel K.R., Brown V.A., Jones D.J., Britton R.G., Hemingway D., Miller A.S., West K.P., Booth T.D., Perloff M., Crowell J.A. (2010). Clinical pharmacology of resveratrol and its metabolites in colorectal cancer patients. Cancer Res.

[129] Boocock D.J., Faust G.E., Patel K.R., Schinas A.M., Brown V.A., Ducharme M.P., Booth T.D., Crowell J.A., Perloff M., Gescher A.J. (2007). Phase I dose escalation pharmacokinetic study in healthy volunteers of resveratrol, a potential cancer chemopreventive agent. Cancer Epidemiol. Biomarkers Prev.

[130] Baur J.A., Sinclair D.A. (2006). Therapeutic potential of resveratrol: The *in vivo* evidence. Nat. Rev. Drug Discov.

[131] Howells L.M., Berry D.P., Elliott P.J., Jacobson E.W., Hoffmann E., Hegarty B., Brown K., Steward W.P., Gescher A.J. (2011). Phase I randomized, double-blind pilot study of micronized resveratrol (SRT501) in patients with hepatic metastases—Safety, pharmacokinetics, and pharmacodynamics. Cancer Prev. Res. (Phila. ).

[132] Mutoh M., Takahashi M., Fukuda K., Komatsu H., Enya T., Matsushima-Hibiya Y., Mutoh H., Sugimura T., Wakabayashi K. (2000). Suppression by flavonoids of cyclooxygenase-2 promoter-dependent transcriptional activity in colon cancer cells: Structure-activity relationship. Jpn. J. Cancer Res.

[133] Priego S., Feddi F., Ferrer P., Mena S., Benlloch M., Ortega A., Carretero J., Obrador E., Asensi M., Estrela J.M. (2008). Natural polyphenols facilitate elimination of HT-29 colorectal cancer xenografts by chemoradiotherapy: A Bcl-2- and superoxide dismutase 2-dependent mechanism. Mol. Cancer Ther.

[134] Kim H.J., Kim S.K., Kim B.S., Lee S.H., Park Y.S., Park B.K., Kim S.J., Kim J., Choi C., Kim J.S. (2010). Apoptotic effect of quercetin on HT-29 colon cancer cells via the AMPK signaling pathway. J. Agric. Food Chem.

[135] Fridrich D., Teller N., Esselen M., Pahlke G., Marko D. (2008). Comparison of delphinidin, quercetin and (−)-epigallocatechin-3-gallate as inhibitors of the EGFR and the ErbB2 receptor phosphorylation. Mol. Nutr. Food Res.

[136] Park C.H., Chang J.Y., Hahm E.R., Park S., Kim H.K., Yang C.H. (2005). Quercetin, a potent inhibitor against beta-catenin/Tcf signaling in SW480 colon cancer cells. Biochem. Biophys. Res. Commun.

[137] Shan B.E., Wang M.X., Li R.Q. (2009). Quercetin inhibit human SW480 colon cancer growth in association with inhibition of cyclin D1 and survivin expression through Wnt/beta-catenin signaling pathway. Cancer Invest.

[138] Chirumbolo S. (2013). Quercetin in cancer prevention and therapy. Integr. Cancer Ther.

[139] Cai X., Fang Z., Dou J., Yu A., Zhai G. (2013). Bioavailability of quercetin: Problems and promises. Curr. Med. Chem.

[140] Li H., Zhao X., Ma Y., Zhai G., Li L., Lou H. (2009). Enhancement of gastrointestinal absorption of quercetin by solid lipid nanoparticles. J. Control Release.

[141] Din F.V., Valanciute A., Houde V.P., Zibrova D., Green K.A., Sakamoto K., Alessi D.R., Dunlop M.G. (2012). Aspirin inhibits mTOR signaling, activates AMP-activated protein kinase, and induces autophagy in colorectal cancer cells. Gastroenterology.

[142] Shpitz B., Giladi N., Sagiv E., Lev-Ari S., Liberman E., Kazanov D., Arber N. (2006). Celecoxib and curcumin additively inhibit the growth of colorectal cancer in a rat model. Digestion.

[143] Lev-Ari S., Strier L., Kazanov D., Madar-Shapiro L., Dvory-Sobol H., Pinchuk I., Marian B., Lichtenberg D., Arber N. (2005). Celecoxib and curcumin synergistically inhibit the growth of colorectal cancer cells. Clin. Cancer Res.

[144] Cruz-Correa M., Shoskes D.A., Sanchez P., Zhao R., Hylind L.M., Wexner S.D., Giardiello F.M. (2006). Combination treatment with curcumin and quercetin of adenomas in familial adenomatous polyposis. Clin. Gastroenterol. Hepatol.

[145] Aftab N., Vieira A. (2010). Antioxidant activities of curcumin and combinations of this curcuminoid with other phytochemicals. Phytother. Res.

[146] Majumdar A.P., Banerjee S., Nautiyal J., Patel B.B., Patel V., Du J., Yu Y., Elliott A.A., Levi E., Sarkar F.H. (2009). Curcumin synergizes with resveratrol to inhibit colon cancer. Nutr. Cancer.

